# Plasma and Erythrocyte Fatty Acid Patterns in Patients with Recurrent Depression: A Matched Case-Control Study

**DOI:** 10.1371/journal.pone.0010635

**Published:** 2010-05-14

**Authors:** Johanna Assies, François Pouwer, Anja Lok, Roel J. T. Mocking, Claudi L. H. Bockting, Ieke Visser, Nico G. G. M. Abeling, Marinus Duran, Aart H. Schene

**Affiliations:** 1 Program for Mood Disorders, Department of Psychiatry, Academic Medical Centre, Amsterdam, The Netherlands; 2 Department of Medical Psychology and Neuropsychology, Center of Research on Psychology in Somatic Diseases (CoRPS), Tilburg University, Tilburg, The Netherlands; 3 Department of Clinical Psychology, University of Groningen, Groningen, The Netherlands; 4 PsyQ Mental Heath Care, Zaandam, The Netherlands; 5 Laboratory Genetic Metabolic Diseases, Academic Medical Centre, Amsterdam, The Netherlands; Mental Health Research Institute of Victoria, Australia

## Abstract

**Background:**

The polyunsaturated fatty acid (PUFA) composition of (nerve) cell membranes may be involved in the pathophysiology of depression. Studies so far, focussed mainly on omega-3 and omega-6 PUFAs. In the present study, saturated fatty acids (SFAs), monounsaturated fatty acids (MUFAs) and PUFAs of the omega-3, -6 and -9 series in plasma and erythrocytes of patients with recurrent major depressive disorder (MDD-R) were compared with controls.

**Methodology and Principal Findings:**

We carried out a case-control study. The sample consisted of 137 patients with MDD-R and 65 matched non-depressed controls. In plasma and erythrocytes of patients with MDD-R the concentrations of most of the SFAs and MUFAs, and additionally erythrocyte PUFAs, all with a chain length >20 carbon (C) atoms, were significantly lower than in the controls. In contrast, the concentrations of most of the shorter chain members (≤18C) of the SFAs and MUFAs were significantly higher in the patients. Estimated activities of several elongases in plasma of patients were significantly altered, whereas delta-9 desaturase activity for C14∶0 and C18∶0 was significantly higher.

**Conclusions/Significance:**

The fatty acid status of patients with MDD-R not only differs with regard to omega-3 and omega-6 PUFAs, but also concerns other fatty acids. These alterations may be due to: differences in diet, changes in synthesizing enzyme activities, higher levels of chronic (oxidative) stress but may also result from adaptive strategies by providing protection against enhanced oxidative stress and production of free radicals.

## Introduction

Major depressive disorder (MDD) -in particular its recurrent, chronic form (MDD-R)- is ranked as a major cause of disability and excess mortality worldwide [1]. Moreover, mortality studies indicate that cardiovascular disease (CVD) accounts for more excess death in MDD patients than any other single cause [2].

Fatty acids (FAs) may play a key role in the pathogenesis of both MDD and CVD and so could explain their mutual association [3]. Reported alterations in FAs in MDD include a low omega (ω)-3 polyunsaturated fatty acid (PUFA) intake, a decrease in ω-3 PUFAs and increased ω-6/ω-3 PUFA ratios in plasma, erythrocytes, adipose tissue and post mortem brain tissue [4], [5], [6]. The nature of these FA alterations still has to be elucidated [7].

PUFAs are essential constituents of the human brain and involved in the regulation of cognition and emotion [8]. PUFAs of the ω-3 and ω-6 series are key components of (nerve) cell membrane phospholipids (PLs) and synapses and are responsible for: signal transduction, ion transport and receptor sensitivity (e.g. for serotonin, dopamine, endocannabinoids) [9]. Important members of the ω-3 and ω-6 series are eicosapentaenoic acid (EPA, C20∶5 ω-3) and arachidonic acid (AA, C20∶4 ω-6) which are precursors for eicosanoids (prostaglandins, leukotrienes, thromboxanes) which mediate infection, inflammation and haemostasis. Importantly, AA derived eicosanoids have a stimulatory effect while EPA derived eicosanoids have a suppressive effect on these processes. Furthermore, docosahexaenoic acid (DHA, C22∶6 ω-3) derived docosanoids (resolvins, neuroprotectins) have a neuroprotective effect [8], [9], [10].

**Figure 1:**
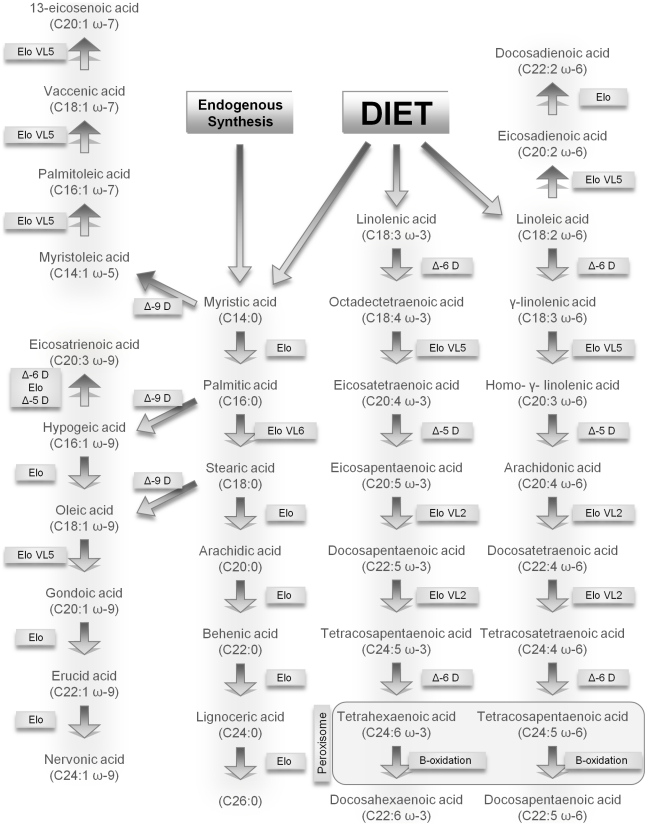
Pathways of fatty acid metabolism. Due to figure dimension restrictions, the conversion of C16∶0 to C16∶1 ω-7 could not be depicted. Abbreviations used: Elongase (Elo), Desaturase (D).

Humans are dependent on their diet for the intake of the two major 18 carbon (C) chain precursors of the ω-3 and ω-6 series of FAs: α-linolenic acid (ALA, C18∶3 ω-3) and linoleic acid (LA, C18∶2 ω-6). Both ALA and LA are transformed by elongases and desaturases to longer chain PUFAs containing three to six double bonds (Fig. 1) [10], [11].

Delta (Δ)6 and Δ5 desaturases introduce double bonds in the ω-3 and ω-6 PUFAs; Δ9 desaturases synthesize monounsaturated fatty acids (MUFAs) from saturated fatty acids (SFAs) [10], [11], [12]. Elongation to long chain (LC) FAs with up to 22 Cs and very long chain (VLC) FAs with >22 Cs occurs in the endoplasmatic reticulum by elongases [11], [13]. Because there is only limited (<15%) conversion of ALA to EPA and DHA and from LA to AA, while the ω-3 and ω-6 PUFAs compete for the same desaturases and elongases, a well balanced ω-6/ω-3 diet is important to reach a sufficient FA status [14]. An ω-6/ω-3 PUFA ratio of <4∶1 is thought to represent a healthy balance. Fatty fish is the major source of ω-3 PUFAs in humans. In Western industrialized countries increases in dietary ω-6 FA intake and reductions in ω-3 FA has changed the ω-6/ω-3 FA ratio to an estimated 15∶1 [15].

Plasma FA levels reflect recent dietary intake while longer-term dietary impact is better reflected in erythrocyte (membrane) and adipose tissue FA composition [16]. Moreover FA status depends not only on dietary intake, but also on endogenous metabolism. The relationship between intake and incorporation into peripheral tissues was found to be non-linear and modulated by genetic factors, age, gender and oxidative stress generated by life style (stress, smoking, use of alcohol, physical activity) [7], [16].

An important limitation of earlier studies on FA levels in MDD is that they mostly addressed only ω-3 and ω-6 PUFA levels. They did not measure the whole FA spectrum, neither were estimates of their respective desaturases and elongases routinely reported [4,5.7]. Moreover, these studies focussed mainly on patients suffering from a single depressive episode while MDD is increasingly considered as a recurrent and often chronic disorder rather than a single episode disorder [17]. Data regarding the FA metabolism of patients with recurrent depression is currently lacking. We hypothesize that -compared to non-depressed controls- patients with MDD-R would have lower PUFA levels and a higher ω-6/ω-3 ratio and that these alterations are “trait” dependent, i.e. independent of the current depressive status.

We previously compared FA levels in plasma and erythrocytes of 44 patients randomly chosen out of a cohort of 137 MDD-R patients with laboratory reference values [18]. We subsequently analyzed the FA spectrum and estimated activities of their respective desaturases and elongases of the whole cohort and compared them with a matched healthy control group (n = 65).

## Materials and Methods

### Population

Recurrently depressed patients participated in a randomized clinical trial comparing the efficacy of preventive cognitive therapy on relapse and recurrence (DELTA Study). The background and methodology have been described elsewhere [17]. In brief, the inclusion criteria of the trial were: age between 18 and 65 years, at least two depressive episodes in the previous 5 years, and having reached current remission status according to the criteria of fourth edition of the Diagnostic and Statistical Manual. Exclusion criteria were current or previous mania or hypomania (bipolar illness), any psychotic disorder (current or previous), organic brain damage, alcohol or drug abuse, predominant anxiety disorder, recent ECT, recent cognitive treatment or receiving CT at the start of the study or current psychotherapy with a frequency of more than two times a month. The medical ethical committee of the Academic Medical Centre approved the study protocol.

At the 2-years follow up of the original trial, the patients participated in the current study, so patients were either in a relapse (depressed) or a remission (non-depressed). Furthermore, we recruited age and gender matched, healthy, non-depressed control subjects by advertising. All participants provided written informed consent prior to enrolment.

### Assessment of depression and other variables

For the current study the psychiatric status was assessed with the Structured Clinical Interview of DSM-IV disorders (SCID-I) by trained interviewers [19]. Information about the use of antidepressants during sampling was obtained by the Trimbos/IMTA questionnaire for Costs associated with psychiatric illness (TIC-P:40) and by interview [20]. Furthermore, blood was sampled in the non fasting state and anthropometric measurements were taken, including: body mass index (BMI; kg/m2), WC in cm and waist to hip ratio (WHR). WC was measured at the level midway between lower rib and the iliac crest, with participants in standing position.

### Blood sample collection and analysis of FAs

FAs in plasma and washed erythrocytes were analyzed by capillary gas chromatography, as described previously [18], [21]. Plasma was separated within 4 h of collection and stored at −80°C until analysis. Total FAs in plasma and washed erythrocytes were expressed as µmol/L and pmol/10e6 erythrocytes respectively. FAs were analyzed both quantitatively and qualitatively. Qualitative analysis of FAs as a percentage of total FAs may be misleading; because the consequence of this approach is that an increase in one FA results in a relative decrease in other FAs to maintain 100% [16]. Therefore we chose to present the results in concentrations. The analysis in percentages is given in supplementary tables (S1, S2). The estimated activities (or surrogate measures) of Δ5, Δ6 and Δ9 desaturases and elongases in plasma are expressed as product/precursor ratios. We do not estimate these activities in erythrocytes because they are reported to be incapable of chain elongation or desaturation of FAs [16].

### Statistical analysis

Statistical analyses were performed using SPSS version 16.0 (SPSS Inc., Chicago, IL, USA). Continuous demographic data of the patients with MDD-R and non-depressed matched controls were analyzed using independent means t-tests and χ^2^ tests were used for categorical variables. Additional tests with correction for WC were performed by the use of ANCOVA's. ANOVA's were also performed in additional, explorative analyses to test whether FA concentrations differed between patients with continuous, intermittent or without use of anti-depressant medication. Independent means t-tests were performed to detect differences in FA concentrations between currently depressed and non-depressed patients.

In FA research, multiple tests have to be performed because of the many members of the different FA series. Therefore the results should be interpreted cautiously with regard to type I errors. We have chosen not to adjust our p-value for multiple testing for the following reasons. First, although our research was partly exploratory (with regard to FA series other than the ω-3 and ω-6), the main part of the analyses (the ω-3 and ω-6 series) was hypothesis driven. Second, it is not common practice in FA research to adjust the p-value for multiple testing. Third, adjustment for multiple testing may induce type II errors.

## Results

Sample characteristics of the patients and the non-depressed control group are displayed in Table 1. Body weight, BMI, WC and WHR were significantly higher in MDD-R patients, compared to non-depressed controls. Patients had a lower education level, smoking habits did not differ. Information on anti-depressant use of the MDD-R patients is given in Table 2.

**Table 1:** Demographic and clinical characteristics of MDD-R patients and the matched non-depressed control group.

	Controls (n = 65)	MDD-R (n = 137)	*p*-value^a^
Age (years ± SD)	44±9	44±10	ns
Male sex (% (n/N))	28% (18/65)	26% (35/137)	ns
Weight (kg ± SD)	73±13	79±16	*
BMI ± SD	24.5±3.5	26.8±5.2	***
WC (cm ± SD)	83.7±12.2	89.3±13.8	**
WHR ± SD	0.81±0.08	0.85±0.08	**
Smoking (% (n/N))	22.6% (14/62)	31.0% (35/113)	ns
Education (% (high/middle/low))	74.6/20.3/5.1	35.3/30.9/33.8	***
Current depression (%)	0% (0/65)	19% (26/136)	***

a Independent means t-tests or χ^2^ tests: significantly different in comparison to controls at * p<.05, ** p<.01, *** p<.001.

MDD-R, Major Depressive Disorder, Recurrent form; BMI, Body Mass Index; WC, Waist Circumference; WHR, Waist-to-Hip Ratio.

**Table 2:** Anti-depressant use of MDD-R patients.

	Subcategory	Result (% (n/N))
Current use of anti-depressants		63% (81/129)
Type of current anti-depressant	TCA	9% (7/81)
	SSRI	65% (53/81)
	SNRI	19% (15/81)
	Antidepressant and/or lithium	6% (5/81)
	Other	1% (1/81)

TCA, Tricyclic Anti-Depressants; SSRI, Selective Serotonin Reuptake Inhibitors; SNRI, Serotonine Noradrenaline Reuptake Inhibitors.

### FA concentrations in plasma (Table 3)

The sums of total FAs, SFAs, MUFAs and PUFAs were all significantly (p<.001) higher in the recurrently depressed patients than in the non-depressed control subjects.

**Table 3:** Plasma fatty acid concentrations (µmol/l) of MDD-R patients compared with a matched non-depressed control group^a^.

	Controls (n = 65)	MDD-R (n = 137)
Linolenic acid (C18∶3 ω-3)	63±40	79±45*
Octadectetraenoic acid (C18∶4 ω-3)	1.5±1.9	1.4±2.6
Eicosapentaenoic acid (C20∶5 ω-3)	67±44	73±44
Docosapentaenoic acid (C22∶5 ω-3)	34±11	31±12
Docosahexaenoic acid (C22∶6 ω-3)	130±53	125±47
**Σ omega-3 PUFAs**	295±110	309±111
Linoleic acid (C18∶2 ω-6)	3040±685	3629±894***
Gamma-linolenic acid (C18∶3 ω-6)	47±20	57±34*
Homogamma linolenic acid (C20∶3 ω-6)	156±94	159±65
Arachidonic acid (C20∶4 ω-6)	554±165	572±162
Docosatetraenoic acid (C22∶4 ω-6)	14±5	14±6
Docosapentaenoic acid (C22∶5 ω-6)	9±4	9±5
Eicosadienoic acid (C20∶2 ω-6)	19±10	21±7
Docosadienoic acid (C22∶2 ω-6)	0.0±0.0	5.2±7.2***
**Σ omega-6 PUFAs**	3840±785	4481±1015***
Myristoleic acid (C14∶1 ω-5)	8.8±7.5	21.3±23.5***
Palmitoleic acid (C16∶1 ω-7)	242±124	388±280***
Vaccenic acid (C18∶1 ω-7)	163±46	198±63***
13-eicosenoic acid (C20∶1 ω-7)	13±7	9±21
**Σ omega-7 PUFAs**	418±168	595±333***
Hypogeic acid (C16∶1 ω-9)	42±15	55±23***
Oleic acid (C18∶1 ω-9)	1975±722	2439±1033***
Gondoic acid (C20∶1 ω-9)	14±6	15±8
Erucid acid (C22∶1 ω-9)	12±8	4±7***
Nervonic acid (C24∶1 ω-9)	64±20	54±12***
Eicosatrienoic acid (C20∶3 ω-9)	10±5	10±7
**Σ omega-9 PUFAs**	2110±754	2578±1057***
Myristic acid (C14∶0)	146±74	213±163***
Pentadecanoic acid (C15∶0)	30±9	34±16
Palmitic acid (C16∶0)	2651±707	3163±1271***
Stearic acid (C18∶0)	741±174	787±242
Arachidic acid (C20∶0)	25.5±6.1	28.5±7.9**
Behenic acid (C22∶0)	58±12	53±13**
Lignoceric acid (C24∶0)	38±8	37±9
**Σ Saturated fatty acids** b	3660±942	4297±1642***
**Σ Monounsaturated fatty acids**	2527±881	3183±1365***
**Σ Polyunsaturated fatty acids**	4145±837	4801±1077***
**Σ Total fatty acids**	10382±2442	12273±3836***

a Independent means t-tests: significantly different in comparison to controls at * p<.05, ** p<.01, *** p<.001.

b C15∶0 excluded due to missing values.

The sum of the FAs of the ω-3 series in plasma did not differ between patients and controls; the concentration of the precursor C18∶3 ω-3, was higher (p<.05), while levels of EPA and DHA were similar in patients and controls. The sum of ω-6 PUFAs was significantly higher in patients (p<.001) and the concentration of the ω-6 series precursor linoleic acid (C18∶2 ω-6) and the next member (C18∶3 ω-6) were significantly higher in the patients (p<.001, p<.05). The concentrations of other members of the ω-6 series did not differ significantly between patients and controls.

The concentration of the MUFA C14∶1 ω-5 was significantly higher in patients' plasma than in controls (p<.001). The sum of ω-7 MUFAs was significantly higher in the patients (p<.001) as were the first two members of the ω-7 series, C16∶1 ω-7 and C18∶1 ω-7, but C20∶1 ω-7 was similar in patients and controls. The sum of ω-9 MUFAs and the first two members C16∶1 ω-9 and C18∶1 ω-9 were all significantly higher in the patients than in controls (p<.001).The concentration of C20∶1 ω-9 was similar, but levels of C22∶1 ω-9 and C24∶1 ω-9 were both significantly lower in the patients (p<.001).

The plasma SFAs concentrations of C14∶0 and C18∶0 were both significantly higher (p<.001), but concentrations of C20∶0 and C22∶0 were significantly lower in patients than in controls (p<.01).

### FAs in erythrocytes (Table 4)

Compared to healthy controls, the sums of total FAs and PUFAs were significantly (p<.001) lower in the MDD-R patients, while the sums of SFAs and MUFAs were similar.

**Table 4:** Erythrocyte fatty acid concentrations (pmol/10e6 erythrocytes) of MDD-R patients compared with a matched non-depressed control group^a^.

	Controls (n = 65)	MDD-R (n = 137)
Linolenic acid (C18∶3 ω-3)	0.81±0.32	0.84±0.31
Octadectetraenoic acid (C18∶4 ω-3)	0.035±0.11	0.21±0.27***
Eicosapentaenoic acid (C20∶5 ω-3)	3.9±2.0	3.3±1.6
Docosapentaenoic acid (C22∶5 ω-3)	10.5±2.1	7.9±1.5***
Docosahexaenoic acid (C22∶6 ω-3)	20.1±6.6	14.8±4.2***
**Σ omega-3 PUFAs**	35.3±10	26.9±6.4***
Linoleic acid (C18∶2 ω-6)	67±12	66±13
Gamma-linolenic acid (C18∶3 ω-6)	0.38±0.37	0.57±0.21***
Homogamma linolenic acid (C20∶3 ω-6)	9.7±2.1	8.8±2.4*
Arachidonic acid (C20∶4 ω-6)	81.6±8.7	71.5±10.5***
Docosatetraenoic acid (C22∶4 ω-6)	13.0±2.5	10.7±2.6***
Docosapentaenoic acid (C22∶5 ω-6)	2.1±0.6	1.7±0.6***
Eicosadienoic acid (C20∶2 ω-6)	1.3±0.4	1.3±0.4
Docosadienoic acid (C22∶2 ω-6)	0±0	0.37±0.43***
**Σ omega-6 PUFAs**	175±19.9	161.4±19.0***
Myristoleic acid (C14∶1 ω-5)	0.60±0.66	0.25±0.31***
Palmitoleic acid (C16∶1 ω-7)	2.5±1.3	3.0±1.5*
Vaccenic acid (C18∶1 ω-7)	7.9±1.4	7.5±1.5
13-eicosenoic acid (C20∶1 ω-7)	0.27±0.35	0.21±0.29
**Σ omega-7 PUFAs**	10.6±2.3	10.6±2.5
Hypogeic acid (C16∶1 ω-9)	1.9±3.0	0.9±0.6*
Oleic acid (C18∶1 ω-9)	75±10	74±10
Gondoic acid (C20∶1 ω-9)	1.2±0.4	1.2±0.4
Erucid acid (C22∶1 ω-9)	2.1±1.4	1.9±2.2
Nervonic acid (C24∶1 ω-9)	19.6±3.5	13.3±3.4***
Eicosatrienoic acid (C20∶3 ω-9)	0.31±0.24	0.36±0.24
**Σ omega-9 PUFAs**	100±13	92±10***
Myristic acid (C14∶0)	3.5±1.2	3.3±1.0
Palmitic acid (C16∶0)	156±19	164±23*
Stearic acid (C18∶0)	104±11	103±11
Arachidic acid (C20∶0)	2.8±0.5	2.5±0.4***
Behenic acid (C22∶0)	9.5±1.6	7.6±1.5***
Lignoceric acid (C24∶0)	21.3±3.0	14.8±4.2***
**Σ Saturated fatty acids**	297±32	295±29
**Σ Monounsaturated fatty acids**	112±14	112±14
**Σ Polyunsaturated fatty acids**	211±23	189±18***
**Σ Total fatty acids**	620±66	588±54***

a Independent means t-tests: significantly different in comparison to controls at * p<.05, ** p<.01, *** p<.001.

The sum of ω-3 PUFAS in erythrocytes was significantly lower in patients than in controls (p<.001). The ω-3 precursor C18∶3 ω-3 did not differ but its Δ6 desaturase product C18∶4 ω-3 was significantly higher in patients than in controls (p<.001). EPA concentrations were not different but C22∶5 ω-3 -the elongase product of EPA- and DHA were both significantly lower in the patients (p<.001).

Analogous to the sum of ω-3 PUFAs, the sum of ω-6 PUFAs in the erythrocytes was also significantly lower in the patients than in the control subjects (p<.001). The concentration of the precursor C18∶2 ω-6 was similar, while the concentration of its Δ6 desaturase product C18∶3 ω-6 was significantly higher in patients than in controls (p<.001). The subsequent members of the ω-6 series: AA, C22∶4 ω-6 and C22∶5 ω-6 were all significantly lower (p<.001) in the patients.

The concentration of the erythrocyte MUFA C14∶1 ω-5 was significantly lower in the patients than in the control subjects (p<.001). In the ω-9 series C24∶1 ω-9 (nervonic acid) and the sum of the ω-9 FAs were significantly lower in the patients (p<.001).

The levels of SFAs C14∶0 and C18∶0 were similar but levels of C20∶0, C22∶0 and C24∶0 were all significantly lower in the patients (p<.001).

### Ratios, desaturases and elongases (Table 5, 6, 7)

The various ratios between the sums of SFA, MUFAs, PUFAs, ω-3 and ω-6 PUFAs and AA/EPA and AA/DHA in the plasma did not differ between the patients and control subjects (Table 5). In the erythrocyte, the ratios of the Σ SFA/Σ PUFA, the Σ MUFA/Σ PUFA, and Σ SFA/Σ MUFA and the Σ ω-6/Σ ω-3 PUFAs were higher in the patients but the AA/EPA and the AA/DHA ratios did not differ (Table 6).

**Table 5:** Several ratios of fatty acids concentrations in plasma of MDD-R patients compared with a matched non-depressed control group^a^.

	Controls (n = 65)	MDD-R (n = 137)
Σ SFAs/Σ PUFAs	1.154±0.169	1.172±0.232
Σ MUFAs/Σ PUFAs	0.603±0.147	0.652±0.178
Σ SFAs/Σ MUFAs	0.885±0.143	0.889±0.202
Σ omega-6/Σ omega-3	14.37±4.59	15.79±4.80
C20∶4 ω-6/C20∶5 ω-3	11.56±7.38	10.58±6.20
C20∶4 ω-6/C22∶6 ω-3	4.887±2.366	5.010±1.7062

a Independent means t-tests: significantly different in comparison to controls at * p<.05, ** p<.01, *** p<.001.

**Table 6:** Several ratios of fatty acids concentrations in erythrocytes of MDD-R patients compared with a matched non-depressed control group^a^.

	Controls (n = 65)	MDD-R (n = 137)
Σ SFAs/Σ PUFAs	1.414±0.065	1.572±0.128***
Σ MUFAs/Σ PUFAs	0.529±0.043	0.548±0.057*
Σ SFAs/Σ MUFAs	2.684±1.773	2.884±0.232***
Σ omega-6/Σ omega-3	5.437±2.363	6.377±1.773**
C20∶4 ω-6/C20∶5 ω-3	27.45±16.79	26.41±12.71
C20∶4 ω-6/C22∶6 ω-3	5.149±5.863	5.232±1.701

a Independent means t-tests: significantly different in comparison to controls at * p<.05, ** p<.01, *** p<.001.

**Table 7:** Several indices of desaturases and elongases in plasma of MDD-R patients compared with a matched non-depressed control group^a^.

	Controls (n = 65)	Delta (n = 137)
Delta-6 omega-3 (C18∶4n-3/C18∶3n-3)	0.029±0.039	0.023±0.039
Delta-5 omega-3 (C20∶5n-3/C18∶4n-3)	25.25±17.56	26.96±21.6
Delta-6 omega-3 (C22∶6n-3/C22∶5n-3)	3.99±1.44	4.33±1.49
Elongase (C22∶5n-3/C20∶5n-3)	0.66±0.34	0.52±0.25**
Delta-6 omega-6 (C18∶3n-6/C18∶2n-6)	0.016±0.007	0.016±0.008
Delta-5 omega-6 (C20∶4n-6/C20∶3n-6)	3.94±1.22	3.95±1.45
Delta 6 omega-6 (C22∶5n-6/C22∶4n-6)	0.67±0.16	0.65±0.22
Elongase (C20∶3n-6/C18∶3n-6)	3.81±2.67	3.47±3.05
Elongase (C22∶4n-6/C20∶4n-6)	0.025±0.007	0.024±0.007
Delta-9 (C14∶1n-5/C14∶0)	0.052±0.032	0.103±0.114***
Delta-9 (C16∶1n-7/C16∶0)	0.02±0.003	0.02±0.02
Delta-9 (C18∶1n-9/C18∶0)	2.64±0.55	3.10±0.77***
Delta-9 (C24∶1n-9/C24∶0)	1.714±0.485	0.529±0.380**
Elongase (18∶∶1 n-9/C16∶1 n-9)	54.20±47.88	55.02±64.23
Elongase (C20∶1 n-9/C18∶1n-9)	0.007±0.002	0.007±0.006
Elongase (22∶1 n-9/C20∶1 n-9)	0.90±0.71	0.33±0.61***
Elongase (C24∶1 n-9/C22∶1n-9)	7.70±6.56	13.62±6.90***
Elongase (C18∶1n-7/C16∶1n-7)	0.77±0.25	0.65±0.28**
Elongase (C20∶1n-7/C18∶1n-7)	0.08±0.04	0.05±0.10*
Elongase (C16∶0/C14∶0)	20.62±6.26	18.26±6.54*
Elongase (C18∶0/C16∶0)	0.28±0.04	0.28±0.29
Elongase (C20∶0/C18∶0)	0.035±0.05	0.037±0.009*
Elongase (C22∶0/C20∶0)	2.32±0.36	1.97±0.57***
Elongase (C24∶0/C22∶0)	0.65±0.069	0.71±0.12***

a Independent means t-tests: significantly different in comparison to controls at * p<.05, ** p<.01, *** p<.001.

The estimated activities of desaturases and elongases in plasma are given in Table 7. Δ6 and Δ5 desaturases did not differ in their activity. The estimated activity of the Δ9 desaturases (C14∶1 ω-5/C14∶0 and C18∶1 ω-9/C18∶0) was significantly higher in patients than in the control subjects (p<.001), but C24∶1 ω-9/C24∶0 was significantly lower (p<.01).

In the ω-3 series the estimated activity of the elongase C22∶5 ω-3/C20∶5 ω-3 was significantly lower in the patients with MDD-R (p<.01). In the ω-9 series, the activity of the elongase C22∶1 ω-9/ C20∶1 ω-9 was significantly lower, but the activity of the next elongase C24∶1 ω-9/C22∶1 ω-9 was significantly higher in the patients compared to controls (p<.001). The estimated activities of elongases in the ω-7 series (C18∶1 ω-7/C16∶1 ω-7 and C20∶1 ω-7/C18∶1 ω-7) were significantly lower in the patients. The estimated activity of elongase C22∶0/C20∶0 was significantly lower and the elongase C24∶0/C22∶0 significantly higher in the patients (p<.001).

### Adjustment for waist circumference (Table 8)

Adjustment for WC as a characteristic of the Metabolic Syndrome (MetS), did not alter the significance of the differences between the concentrations of the FAs in the erythrocytes of the patients compared to the controls [We divided the p-values in four categories (p≥0.05, p<0.05, p<0.01, p<0.001); no altering of significance means the p-value remained in the same category]. In plasma, the significance was altered for the differences in the concentrations of the following plasma FAs [C14∶0, C16∶0, C20∶0, C16∶1 ω-7, C18∶1 ω-7, C16∶1 ω-9, C18∶1 ω-9 and C18∶3ω-3 (ALA)] between patients with MDD-R and controls (Table 8).

**Table 8:** Plasma fatty acid concentrations (µmol/l) of MDD-R patients compared with a matched non-depressed control group, with and without correction for waist circumference^a^.

	Controls^b^ (n = 65)	MDD-R^b^ (n = 137)	Controls (n = 65)	MDD-R (n = 137)
Linolenic acid (C18∶3 ω-3)	67.38±5.6	77.22±3.9	63±40	79±45*
Palmitoleic acid (C16∶1 ω-7)	267.3±29.7	375.2±20.6**	242±124	388±280***
Vaccenic acid (C18∶1 ω-7)	169.5±7.32	195.6±5.08**	163±46	198±63***
Hypogeic acid (C16∶1 ω-9)	43.70±2.63	54.43±1.82**	42±15	55±23***
Oleic acid (C18∶1 ω-9)	2086±116.5	2389±80.80*	1975±722	2439±1033***
Myristic acid (C14∶0)	157.4±17.9	207.1±12.4	146±74	213±163***
Palmitic acid (C16∶0)	2794±138.1	3105±95.76	2651±707	3163±1271***
Arachidic acid (C20∶0)	26.2±0.96	28.3±0.66	25.5±6.1	28.5±7.9**

a ANCOVA's with correction for WC on the left and independent means t-tests on the right: significantly different in comparison to controls at * p<.05, ** p<.01, *** p<.001.

b WC corrected values.

### Influence of anti-depressant use

To explore whether use of antidepressants was associated with different FA patterns we performed explorative ANOVA's, comparing three groups of patients: 1) those who had used antidepressants continuously, 2) intermittent or 3) not at all. No differences in FA concentrations between these groups were found, except for C22∶5 ω-3 in plasma (resp. 30.94 vs. 27.47 vs. 34.26, p<0.05) and C18∶3 ω-3 (0.97 vs. 0.75 vs. 0.83, p<0.05) and 20∶5 ω-3 (3.99 vs. 2.92 vs. 3.29, p<0.05) in erythrocyte membranes.

### Influence of current depressive status

In the group of patients with recurrent depression, and FA concentrations of the patients with a current depression (n = 26) were compared with those of the non-depressed (n = 110) using explorative t-tests. The results showed no significant differences in mean plasma and erythrocyte FA concentrations between the two groups.

### Influence of education level

After correction for differences in educational level (low, middle, high), the difference in C18∶3 ω-3 in plasma between patients and controls, disappeared (67±6.3 vs. 78±4.2, p = .164). The other FAs in plasma and erythrocytes were not altered.

## Discussion

Previous studies generally focussed on ω-3 and ω-6 PUFAs in samples of patients with a single depressive episode or high levels of depressive symptoms [4], [5], [7]. In the present study we were able to compare not only PUFA concentrations of the ω-3 and ω-6 series, but also ω-5, ω-7 and ω-9 MUFAs, as well as SFAs, in plasma and erythrocytes of patients with the recurrent type of MDD with those of matched control subjects.

The results of the present study confirm the results of our explorative pilot study [18]. Most striking was the finding that the concentrations of plasma and erythrocyte MUFAs and SFAs and additionally erythrocyte PUFAs, with a chain length >20 C atoms were all lower in the patients with MDD-R with the exception of C24;0 in the plasma and C22∶1 ω-9 in the erythrocyte which did nor differ in patients and controls. In contrast, the concentrations of most of the shorter chain members (≤18 C) of all these families were higher in patients, with the exception of lower concentrations of C14∶1 ω-5 and C16∶1 ω-9 in erythrocytes.

Plasma FA levels reflect the combined effect of recent intake and endogenous processing [16]. So the increase in SFAs and MUFAs with ≤18 C in the patients may reflect the effect of a higher intake whether or not associated with higher activity of Δ9 desaturases. The estimated activities of the Δ9 desaturases C14∶0/C14∶1 ω-5 and C18∶0/C18∶1 ω-9 in the plasma were significantly higher in the depressive patients (Table 7). Enhanced Δ-9 desaturase activity has also been reported in patients with the MetS [12].

The Σ ω-6 PUFAs in plasma was significantly higher in the patients than in the controls consistent with a higher intake of mainly LA. However, in contrast to many earlier studies, we found that levels of AA, EPA, DHA and the Σ ω-3 PUFAs in plasma were similar in patients and controls, as were AA/EPA and AA/DHA ratios [4], [5], [7]. Also estimated activities of Δ5 and Δ6 desaturases did not differ.

In the erythrocytes of our MDD-R patients the sums of ω-3 and the ω-6 PUFAs were lower and the ratios Σ ω-6/Σ ω-3 were higher than in controls corresponding with many reported data [4], [5], [7]. But as in plasma, the ratios AA/EPA and AA/DHA did not differ between patients and controls. The increase in the Δ6 desaturase products C18∶4 ω-3 and C18∶3 ω-6 might be compensatory to the decreases in C22∶5 ω-3, C22∶6 ω-3 and C20∶3 ω-6 up to C22∶5 ω-6. These alterations are also seen in the MetS [14], [22].

So, part of our most marked findings may be explained by the fact that based on plasma levels, the patients with MDD-R consume more SFAs and MUFAs and ω-6 PUFAs, C18∶2 ω-6 (LA) in particular. The increase in the levels of C22∶2 ω-6 (docosadienoic acid) in plasma and erythrocytes of the patients may also be explained thereby [23].

To our knowledge, concentrations of the longer chain FAs (≥20–24 C) of SFAs and MUFAs and estimates of their elongases were not systematically addressed in patients with MDD. We found a significant decrease of the >20 C plasma SFAs and MUFAs and of all the >20 C erythrocyte FA levels, with the exception of plasma C24∶0 and erythrocyte C22∶1 ω-9 which were similar in patients and controls.

One explanation may be altered elongase activity (Table 7). Interestingly, our results show decreased elongase activity on 20C FAs, whereas elongases acting on 22C FAs show increased activity, with the exception of the ω-6 series. However, the use of FA ratio's for enzyme activity estimation gives only “surrogate” measures and may not reflect real activity [16].

Although not often reported, VLC SFAs and MUFAs, mainly C24∶0 (lignoceric acid) and C24∶1 ω-9 comprise 1–6% of total plasma PLs. These fatty acids are very abundant (up to 46 mol%) in sphingomyelin. In lower concentration (approximately 0.11–0.015) are FAs with 26 carbons such as C26∶0 and C26∶1 ω-9 and C26∶2 ω-6. These fatty acids are also typically found in the sphingomyelin fraction [16].

We would like to hypothesize that the decreases in the longer chain FAs could be related to the use of these longer chain FAs for increased ceramide and sphingolipid synthesis as seen in insulin resistance and MetS [24]. Furthermore, these long chain SFAs could be used for production of C26∶0. High levels of C26∶0 in whole blood were recently shown to be associated with the MetS in Japanese men [25].

Taken together, the cause of the decrease in concentrations of plasma and erythrocyte MUFAs and SFAs and additionally erythrocyte PUFAs, all with a chain length of ≥20 C atoms may well be multifactorial, that is to say modulated by dietary factors, life style, decreased or impaired activity of their respective desaturases and elongases, but could also correspond with decreased or increased incorporation in and/or enhanced detachment from the various plasma lipid components and/or cell membranes. Further research is needed to differentiate between these possibilities.

Consistent with the literature on obesity in depressive disorders, BMI, WHR and WC were all significantly higher in our MDD-R patients than in the control subjects [26]. The MetS criterion of an increased WC (≥88 cm in women and ≥102 cm in men) was met in 44 of the 102 female patients (mean 87; range 63–122). Adjustment for WC, as a peripheral indicator of insulin resistance associated with visceral fat, resulted in reduced significances of the differences in most of the short chain members of the FA series in plasma (Table 8). This may correspond with the presence of the MetS and/or associated factors such as the dietary composition. However, the differences found in the longer chain plasma FAs and all differences in the erythrocyte were not altered by adjustment for WC. So, our main finding of lower concentrations of the longer members of the various FA series can not merely be explained by the presence of the MetS, and may reflect other MDD-R associated factors.

In this study after correction for differences in educational level (low, middle, high), the difference in C18∶3 ω-3 in plasma between patients and controls disappeared, but the other FAs in plasma and erythrocytes were not altered. The percentage of smokers vs. non-smokers did not differ between patients and controls and so are not likely to be responsible for the differences in FA concentrations.

Antidepressant (AD) use was accompanied by altered concentrations of a small number of ω-3 PUFAs (C18∶3 ω-3 and C20∶3 ω-3 in plasma and C22∶5 ω-3 in the erythrocyte membrane), other FA concentrations were not altered. There may be several explanations such as: the presence of a more serious depression in the AD users, the consumption of more food and less physical activity as well as an effect of AD on FA metabolism and (oxidative) stress. To our knowledge data of the direct effects of AD on FAs are still lacking and reports on the effect of AD on oxidative stress are sparse and still inconsistent [27]. Our study was not directed at these aspects so we refrain from drawing conclusions.

We found no influence of current depressive status on FA concentrations, so they seem to be state-independent. This may indicate that the FA alterations in this study could represent a biological “trait” marker for recurrent depression (MDD-R).

An essential characteristic of MDD-R is the presence of increased oxidative stress, which may also modulate FA alterations in our patients. Oxidative stress is the result of an imbalance between excessive free radical oxygen species (ROS) production and/or diminished anti-oxidant defence mechanisms. Evidence for a causal role of oxidative stress in the pathogenesis of psychiatric diseases including bipolar disorder and depression is steadily accumulating [28]. The brain is particularly vulnerable because of its high oxygen consumption and hence generation of ROS combined with a high PUFA content and modest antioxidant defences [29].

Enhanced oxidative stress in our patients may be the result of a cumulative effect of the presence of the MetS and depression associated factors such as: psychological stress, hypothalamic-pituitary-adrenal (HPA)-axis hyperactivity, life style changes (ω-3 PUFA deficient diet, physical inactivity, alcohol abuse and smoking). Major depressive disorder -its recurrent, chronic form in particular- may be accompanied by sustained activation of the HPA-axis [30], [31].

Moreover, mitochondria are the principal ROS producers and evidence for genetically determined mitochondrial dysfunction in psychiatric disease, MDD included, is also growing steadily [32], [33]. Mitochondrial dysfunction will further enlarge increased ROS production and may also explain the increased prevalence of the MetS and CVD in MDD-R.

Noteworthy, the pattern of FA alterations in our patients are not specific for MDD-R but is also found in other (psychiatric) diseases accompanied by increased oxidative stress e.g. bipolar disorder, schizophrenia, diabetes, Alzheimer's disease and are also seen during normal aging [34], [35], [36], [37].

Finally, the FA alterations in MDD-R could fulfil an adaptive or protective role, as the alterations may render cell membranes less vulnerable for oxidation. SFAs and MUFAs are more resistant to oxidative stress, while the more polyunsaturated a FA, the more susceptible it is [38].

Our study has several distinct limitations: dietary intake of fat and FAs, alcohol consumption, smoking habits, physical activity and the presence of the MetS were not systematically assessed. We used ratios for estimating desaturases and elongases and did not measure enzyme expression/activities. However, in spite of these many limitations, our results were quite consistent which may argue for their validity. This consistency, together with the magnitude of our findings, also makes type I errors -that could have occurred because of multiple testing- less likely.

At this moment the basal question; whether fatty acids alterations in depressive patients are either the cause or the consequence of the disease, cannot yet be answered on the basis of available studies (ecologic, observational, and RCTs). They provide inconsistent data, the very few prospective studies included [39], [40]. In their most recent review, Appleton et al. stressed that in relation to the effects of ω-3 PUFAs, an important distinction may exist between diagnosed depressive illness and the less severe, undiagnosed, or precursor “depressed mood” [40].

The best methods to study FAs have not yet crystallized. The FA composition of the different blood lipid fractions, e.g. plasma, erythrocytes and platelets is inter-related, particularly for PLs. As erythrocytes have a life span of approximately 120 days, large changes that occur within days of altering dietary fat intake can only be explained by exchange and transfer of FA from plasma to erythrocytes [16].

Although erythrocyte FA composition is comparable to that of plasma total PLs, there are differences [16]. Given these differences it will prove to be more informative to analyse FAs in both compartments. For prospective studies over several years FA biomarkers from erythrocytes or adipose tissue, which reflect longer-term intakes (preceding months or years, respectively) would be more suitable to test the association between long term PUFA status and depression [39], [40].

Adequately powered intervention studies are urgently needed, studying the effect dependent on the background FA status and the change during the study period. Further FA research should include: dietary questionnaires, analysis of the complete FA spectrum and also the enzymes involved in their metabolism. It is increasingly demonstrated that polymorphisms in the genes/enzymes (desaturases, elongases) that regulate biosynthesis of EPA, DHA and AA from their precursors (ALA, EPA) may represent important determinants of plasma and tissue PUFA levels [41]. Measurement of (oxidative) stress levels and lipid peroxidation products and assessment of MetS symptoms will help us to distinguish between harmful or adaptive FA changes. It will also help us to determine whether patients will benefit from anti-oxidant strategies and/or any form of FA supplementation.

## Supporting Information

Table S1 Plasma fatty acid concentrations (% of total fatty acids) of MDD-R patients compared with a matched non-depressed control group^a^. ^a^Independent means t-tests: significantly different in comparison to controls at * p<.05, ** p<.01, *** p<.001.
(0.05 MB DOC)

Table S2 Erythrocyte fatty acid percentages (% of total fatty acids) of MDD-R patients compared with a matched non-depressed control group^a^. ^a^Independent means t-tests: significantly different in comparison to controls at * p<.05, ** p<.01, *** p<.001.
(0.05 MB DOC)
