# Supplementary material for: Plasma and Erythrocyte Fatty Acid Patterns in Patients with Recurrent Depression: A Matched Case-Control Study
Source: PLoS One. 2010 May 14;5(5):e10635. doi: 10.1371/journal.pone.0010635 (PMC2871041; doi:10.1371/journal.pone.0010635)
Supplement: Table S1 — Plasma fatty acid concentrations (% of total fatty acids) of MDD-R patients compared with a matched non-depressed control groupa. aIndependent means t-tests: significantly different in comparison to controls at * p<.05, ** p<.01, *** p<.001. (0.05 MB DOC) [file pone.0010635.s001.doc]

**Supplementary table 1.** Plasma fatty acid concentrations (% of total fatty acids) of MDD-R patients compared with a matched non-depressed control group^a^

|  | Controls (n = 65) | MDD-R (n = 137) |
| --- | --- | --- |
| Linolenic acid (C18:3 ω-3) | 0.6 ± 0.2 | 0.6 ± 0.2 |
| Octadectetraenoic acid (C18:4 ω-3) | 0.02 ± 0.02 | 0.01 ± 0.1 |
| Eicosapentaenoic acid (C20:5 ω-3) | 0.7 ± 0.4 | 0.6 ± 0.4 |
| Docosapentaenoic acid (C22:5 ω-3) | 0.33 ± 0.08 | 0.26 ± 0.08*** |
| Docosahexaenoic acid (C22:6 ω-3) | 1.3 ± 0.5 | 1.1 ± 0.4* |
| Linoleic acid (C18:2 ω-6) | 29.3 ± 4.3 | 30.6 ± 5.0 |
| Gamma-linolenic acid (C18:3 ω-6) | 0.5 ± 0.2 | 0.5 ± 0.2 |
| Homogamma linolenic acid (C20:3 ω-6) | 1.5 ± 0.7 | 1.3 ± 0.3* |
| Arachidonic acid (C20:4 ω-6) | 5.3 ± 1.3 | 4.8 ± 1.2* |
| Docosatetraenoic acid (C22:4 ω-6) | 0.13 ± 0.04 | 0.11 ± 0.04 |
| Docosapentaenoic acid (C22:5 ω-6) | 0.09 ± 0.03 | 0.07 ± 0.04** |
| Eicosadienoic acid (C20:2 ω-6) | 0.2 ± 0.1 | 0.2 ± 0 |
| Docosadienoic acid (C22:2 ω-6) | 0.0 ± 0.0 | 0.0 ± 0.0 |
| Myristoleic acid (C14:1 ω-5) | 0.1 ± 0.1 | 0.2 ± 0.2*** |
| Palmitoleic acid (C16:1 ω-7) | 2.4 ± 1.0 | 2.9 ± 1.3*** |
| Vaccenic acid (C18:1 ω-7) | 1.6 ± 0.3 | 1.6 ± 0.3 |
| 13-eicosenoic acid (C20:1 ω-7) | 0.12 ± 0.05 | 0.07 ± 0.13** |
| Hypogeic acid (C16:1 ω-9) | 0.4 ± 0.1 | 0.5 ± 0.1** |
| Oleic acid (C18:1 ω-9) | 18.8 ± 3.2 | 19.3 ± 3.0 |
| Gondoic acid (C20:1 ω-9) | 0.1 ± 0.1 | 0.1 ± 0.1 |
| Erucid acid (C22:1 ω-9) | 0.11 ± 0.08 | 0.04 ± 0.08*** |
| Nervonic acid (C24:1 ω-9) | 0.6 ± 0.2 | 0.5 ± 0.2*** |
| Eicosatrienoic acid (C20:3 ω-9) | 0.1 ± 0.1 | 0.1 ± 0.1 |
| Myristic acid (C14:0) | 1.4 ± 0.5 | 1.6 ± 0.7** |
| Pentadecanoic acid (C15:0) | 0.29 ± 0.06 | 0.15 ± 0.16*** |
| Palmitic acid (C16:0) | 25.6 ± 1.8 | 25.3 ± 3.3 |
| Stearic acid (C18:0) | 7.2 ± 0.7 | 6.4 ± 1.1*** |
| Arachidic acid (C20:0) | 0.3 ± 0 | 0.2 ± 0 |
| Behenic acid (C22:0) | 0.6 ± 0.1 | 0.5 ± 0.1*** |
| Lignoceric acid (C24:0) | 0.4 ± 0.1 | 0.3 ± 0.1*** |
