# Supplementary material for: Plasma and Erythrocyte Fatty Acid Patterns in Patients with Recurrent Depression: A Matched Case-Control Study
Source: PLoS One. 2010 May 14;5(5):e10635. doi: 10.1371/journal.pone.0010635 (PMC2871041; doi:10.1371/journal.pone.0010635)
Supplement: Table S2 — Erythrocyte fatty acid percentages (% of total fatty acids) of MDD-R patients compared with a matched non-depressed control groupa. aIndependent means t-tests: significantly different in comparison to controls at * p<.05, ** p<.01, *** p<.001. (0.05 MB DOC) [file pone.0010635.s002.doc]

**Supplementary table 2.** Erythrocyte fatty acid percentages (% of total fatty acids) of MDD-R patients compared with a matched non-depressed control group^a^

|  | Controls (n = 65) | MDD-R (n = 137) |
| --- | --- | --- |
| Linolenic acid (C18:3 ω-3) | 0.1 ± 0.1 | 0.1 ± 0.1 |
| Octadectetraenoic acid (C18:4 ω-3) | 0.0 | 0.0 |
| Eicosapentaenoic acid (C20:5 ω-3) | 0.6 ± 0.3 | 0.6 ± 0.3 |
| Docosapentaenoic acid (C22:5 ω-3) | 1.7 ± 0.3 | 1.4 ± 0.3*** |
| Docosahexaenoic acid (C22:6 ω-3) | 3.2 ± 1.0 | 2.5 ± 0.8*** |
| Linoleic acid (C18:2 ω-6) | 10.8 ± 1.3 | 11.3 ± 1.6 |
| Gamma-linolenic acid (C18:3 ω-6) | 0.1 ± 0.06 | 0.1 ± 0.04 |
| Homogamma linolenic acid (C20:3 ω-6) | 1.6 ± 0.3 | 1.5 ± 0.4* |
| Arachidonic acid (C20:4 ω-6) | 13.2 ± 1.0 | 12.2 ± 1.6*** |
| Docosatetraenoic acid (C22:4 ω-6) | 1.8 ± 0.5 | 0.3 ± 0.1*** |
| Docosapentaenoic acid (C22:5 ω-6) | 0.4 ± 0.1 | 0.3 ± 0.1*** |
| Eicosadienoic acid (C20:2 ω-6) | 0.2 ± 0.1 | 0.2 ± 0.1 |
| Docosadienoic acid (C22:2 ω-6) | - | 0.1 ± 0.1 |
| Myristoleic acid (C14:1 ω-5) | 0.1 ± 0.1 | 0.1 ± 0.1*** |
| Palmitoleic acid (C16:1 ω-7) | 0.4 ± 0.2 | 0.5 ± 0.2* |
| Vaccenic acid (C18:1 ω-7) | 1.3 ± 0.2 | 1.3 ± 0.2 |
| 13-eicosenoic acid (C20:1 ω-7) | 0.04 ± 0.06 | 0.03 ± 0.05*** |
| Hypogeic acid (C16:1 ω-9) | 0.3 ± 0.5 | 0.2 ± 0.1* |
| Oleic acid (C18:1 ω-9) | 12.1 ± 1.1 | 12.7 ± 1.1 |
| Gondoic acid (C20:1 ω-9) | 0.2 ± 0.1 | 0.2 ± 0.1 |
| Erucid acid (C22:1 ω-9) | 0.3 ± 0.2 | 0.3 ± 0.4*** |
| Nervonic acid (C24:1 ω-9) | 3.2 ± 0.5 | 2.3 ± 0.6*** |
| Eicosatrienoic acid (C20:3 ω-9) | 0.1 ± 0.04 | 0.1 ± 0.04*** |
| Myristic acid (C14:0) | 0.6 ± 0.2 | 0.6 ± 0.2 |
| Palmitic acid (C16:0) | 25.1 ± 1.1 | 27.8 ± 2.1* |
| Stearic acid (C18:0) | 16.9 ± 0.8 | 17.6 ± 0.8 |
| Arachidic acid (C20:0) | 0.5 ± 0.1 | 0.4 ± 0.1*** |
| Behenic acid (C22:0) | 1.5 ± 0.2 | 1.3 ± 0.3*** |
| Lignoceric acid (C24:0) | 3.4 ± 0.4 | 2.6 ± 0.8*** |
